# The reaction of CF_2_Cl_2_ with gas-phase hydrated electrons^†^

**DOI:** 10.1039/c6cp01976e

**Published:** 2016-08-15

**Authors:** Jozef Lengyel, Christian van der Linde, Michal Fárník, Martin K. Beyer

**Affiliations:** a Institut für Ionenphysik und Angewandte Physik, Leopold-Franzens-Universität Innsbruck, Technikerstraße 25, 6020 Innsbruck, Austria; b J. Heyrovský Institute of Physical Chemistry v.v.i., Czech Academy of Sciences, Dolejškova 3, 18223 Prague, Czech Republic

## Abstract

The reaction of dichlorodifluoromethane (CF_2_Cl_2_) with hydrated electrons (H_2_O)_n_^−^ (*n* = 30–86) in the gas phase was studied using Fourier transform ion cyclotron resonance (FT-ICR) mass spectrometry. The hydrated electron reacts with CF_2_Cl_2_, forming (H_2_O)_m_Cl^−^ with a rate constant of (8.6 ± 2.2) × 10^−10^ cm^3^ s^−1^, corresponding to an efficiency of 57 ± 15%. The reaction enthalpy was determined using nanocalorimetry, revealing a strongly exothermic reaction with ΔH_r_(CF_2_Cl_2_, 298 K) = −208 ± 41 kJ mol^−1^. The combination of the measured reaction enthalpy with thermochemical data from the condensed phase yields a C–Cl bond dissociation enthalpy (BDE) ΔH_C–Cl_(CF_2_Cl_2_, 298 K) = 355 ± 41 kJ mol^−1^ that agrees within error limits with the predicted values from quantum chemical calculations and published BDEs.

## Introduction

Since the discovery of the atmospheric ozone hole chlorofluoro- carbons (CFCs) have been recognized as one of the important players in ozone depletion.^1,2^ The most common CFC is dichlorodifluoromethane (CF_2_Cl_2_) that was used in refrigerants due to its high latent heat, non-toxicity and inertness. The other factors in these processes are ice particles in polar stratospheric clouds (PSCs).^3^ Thus the chemistry, which involves not only the CFC in the gas phase, but also the environment of the ice particles, needs to be investigated.

The processes involved in ozone depletion are mostly driven by sunlight. Besides photochemistry on ice particles,^4^ electron-induced reactions have also received significant interest. The most prominent example is dissociative electron transfer (DET) on ice surfaces^5–7^ where electron transfer to CFCs was greatly enhanced by several orders of magnitude upon adsorption on ice. The mechanism was explained by the presence of a ‘self-trapped’, solvated excess electron in a polar medium such as water or ammonia. Based on the large enhancement of Cl^−^ generation from the DET to CFCs on ices, Lu and Sanche^8,9^ proposed the cosmic-ray-driven electron reaction model for ozone depletion, short CRE mechanism, as an additional potential source of Cl^⋅^ radicals. This mechanism initiated a controversial debate.^8,10–15^ Cl^−^ enhancement was also observed in other experiments^16,17^ and it was interpreted as an attachment of low energy secondary electrons to CF_2_Cl_2_ solvated in a polar medium. CF_2_Cl_2_ is then decomposed and Cl^−^ is generated due to tunnelling of the solvated electron. In contrast to this interpretation, the dissociative electron attachment (DEA) cross-sections of mixed CF_2_Cl_2_/NH_3_ clusters did not exhibit any enhancement for electron energies in the range of 0–20 eV.^18^ In condensed phase electrochemistry, DET of carbon–halogen bonds has been extensively studied.^19^ A sticky DET mechanism with the Cl^−^ and ${\text{CCl}}_{3}^{•}$ fragments bound by a solvent cage was established by Pause *et al.* ^20^ for DET of CCl_4_ in *N*,*N* ^′^-dimethylformamide, but the strength of the interaction was found to decrease with increasing polarity of the solvent.^21^

DEA to gas-phase CF_2_Cl_2_ is an efficient process due to the high electron affinity of halogen atoms.^22^ Illenberger *et al.* ^23,24^ observed that DEA to CF_2_Cl_2_ at electron energies close to 0 eV occurs *via* reaction (1), with large cross-sections.

(1) $${\text{CF}}_{2}{\text{Cl}}_{2}+{\text{e}}^{-}\to {\text{CF}}_{2}{\text{Cl}}_{2}{*}^{-}\to {\text{Cl}}^{-}+{\text{CF}}_{2}{\text{Cl}}^{•}$$

However, there are very few free electrons in the atmosphere below 50 km altitude,^25^ because they are quickly captured by abundant molecules, in particular O_2_.^26^ Therefore, in the CRE mechanism it is assumed that the PSC particles are able to stabilize solvated electrons generated by ionizing radiation within a condensed phase particle. If CF_2_Cl_2_ is also condensed in the particle, DET with formation of Cl^−^ may take place. For the liquid phase, it has been shown already in 1971 by pulse radiolysis studies in bulk aqueous solution that thermalized hydrated electrons react rapidly with CFCs to produce Cl^−^ ions.^27^

Previous studies in our group have shown that reductive cleavage of carbon–halogen bonds, *i.e.* DET, competes with the first step of Birch reduction in reactions of chlorobenzene as well as di- and trifluorobenzenes with gas-phase hydrated electrons ${\left({\text{H}}_{2}\text{O}\right)}_{n}^{-}$.^28,29^ In order to test whether fully thermalized solvated electrons are capable of inducing DET in CFCs, we examine the reaction of CF_2_Cl_2_ and hydrated electrons ${\left({\text{H}}_{2}\text{O}\right)}_{n}^{-}$ in the cluster size range *n*= 30–86. We report a systematic study on the reaction kinetics. Applying nanocalorimetry, we extract the reaction enthalpy from the experimental data. In combination with literature thermochemistry from the condensed phase, the C–Cl bond dissociation enthalpy (BDE) of gas-phase CF_2_Cl_2_ is derived and compared with literature values as well as high-level quantum chemical calculations. We discuss the observed results in comparison with DET studies in bulk ice and photo-dissociation experiments in water clusters.

## Experiment

The experiments are performed using a modified Bruker/ Spectrospin CMS47X FT-ICR mass spectrometer, equipped with a 4.7 T superconducting magnet, a Bruker infinity cell, and an APEX III data station.^30,31^ Hydrated electrons ${\left({\text{H}}_{2}\text{O}\right)}_{n}^{-}$ are generated in a home built external source^31,32^ by laser vaporization of a solid zinc target and supersonic expansion of the hot plasma in a helium/water gas pulse.^33,34^ The skimmed ${\left({\text{H}}_{2}\text{O}\right)}_{n}^{-}$ cluster beam is transferred *via* an electrostatic lens system through differential pumping stages into the ultra-high vacuum (UHV) region of the mass spectrometer, with a background pressure below 5 × 10^−10^ mbar, and stored in the ICR cell. CF_2_Cl_2_ is introduced into the UHV region of the mass spectrometer as a gas through a leak valve at constant pressures in the range of 0.5–1.1 × 10^−8^ mbar. The purity of the reactant is checked using electron ionization and high resolution mass spectrometry directly in the ICR cell.

To determine the rate constant, reactions are monitored by recording mass spectra as a function of time. The intensities of reactant and product clusters in the mass spectra are summed over all cluster sizes. While the experiments are conducted at room temperature, the internal temperature of (H_2_O)*_n_* ^−^ clusters is a result of the interplay between radiative heating by black-body radiation and evaporative cooling.^35^ In combination with the caloric curves measured by Hock *et al.* ^36^ this places the internal temperature of the clusters in the range of 90–120 K.

Thermochemistry is investigated using nanocalorimetry.^30,37^ The heat released during the reaction is extracted by quantitative modelling of the average size of reactant and product clusters as a function of time, taking into account blackbody radiation induced dissociation (BIRD).^35,38–41^ The method was introduced by Höckendorf *et al*.^30^ in reactions of ${\left({\text{H}}_{2}\text{O}\right)}_{n}^{-}$ with O_2_ and CO_2_.

To extract the reaction enthalpy from the mass spectra, the average cluster size of reactant and product species is calculated. The results are fitted with a genetic algorithm with the following differential equations:

(2) $$\text{d}N_{\text{R}}=-k_{\text{f}}(N_{\text{R}}-N_{\text{0,R}})\text{d}t$$

(3) $$\text{d}N_{\text{P}}=-k_{\text{f}}(N_{\text{P}}-N_{\text{0,P}})\text{d}t+(N_{\text{R}}-\Delta N_{\text{vap}}-N_{\text{P}})\left(\frac{kI_{\text{R}}}{I_{\text{P}}}\right)\text{d}t$$

Eqn (2) and the first term in eqn (3) describe BIRD of water clusters, with *k*_f_ describing the linear dependence on cluster size. *N*_0,R_, *N*_0,P_ account for the contribution of the ionic core to the IR absorption cross-sections. The second term in eqn (3) describes the evaporation of water molecules due to the reaction enthalpy released in the water cluster. The average number of evaporated water molecules Δ*N*_vap_ is the key result of the fit.

The experiments are assisted by quantum chemical calculations at the Gaussian-4 (G4) level^42^ using the Gaussian09 program package^43^ to support the experimentally observed BDE of the CF_2_Cl_2_ molecule. In general, G4 level calculations exhibit an average absolute deviation from experiment of 3.5 kJ mol^−1^.

## Results

**Fig. 1:**
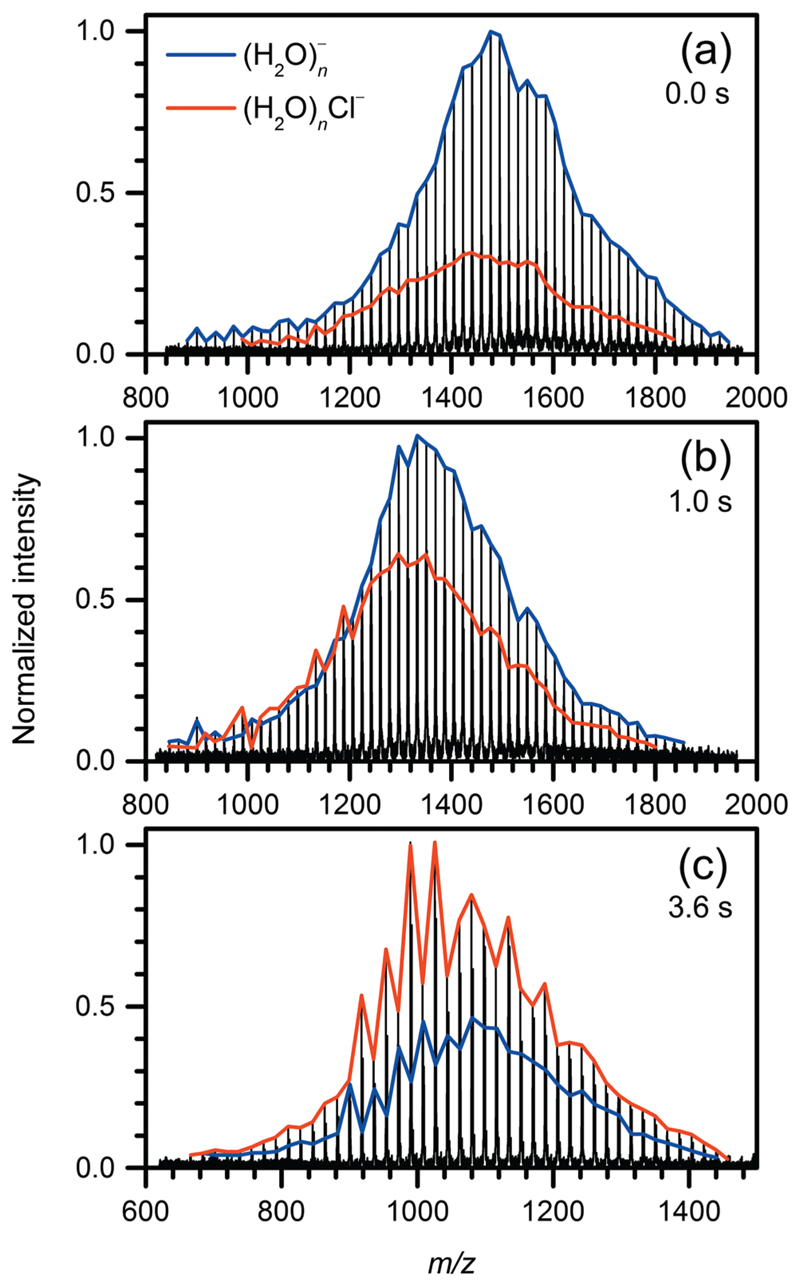
Mass spectra of the reaction of CF_2_Cl_2_ with hydrated electrons (blue line) after (a) 0.0, (b) 1.0, and (c) 3.6 s. (H_2_O)_*m*_Cl^−^ (red line) as the product is present already at nominal 0.0 s due to the 2 s filling cycle.

Fig. 1 shows the mass spectra of the reaction of CF_2_Cl_2_ with ${\left({\text{H}}_{2}\text{O}\right)}_{n}^{-}$. The reaction leads to the formation of hydrated chloride ions and CF_2_Cl^**˙**^ radicals, which evaporate from the water cluster, reaction (4).

(4) $${\text{CF}}_{\text{2}}{\text{Cl}}_{\text{2}}+{\left({\text{H}}_{2}\text{O}\right)}_{n}^{-}\to {\left({\text{H}}_{2}\text{O}\right)}_{m}{\text{Cl}}^{-}+{\text{CF}}_{\text{2}}{\text{Cl}}^{•}+\left(n-m\right){\text{H}}_{2}\text{O}$$

At initial 0 s, the mass spectrum is dominated by hydrated electrons. However, a small amount of the product ions is present. Some clusters have reacted during the accumulation in the ICR cell, which takes 2 s. The intensity of the product ions increases with the delay time. After 1 s, the product ions represent more than 50% of the hydrated electron intensity. The product ions start to dominate after 1.5 s. At longer times, the shift of the cluster size distribution to smaller values is clearly visible. Both species undergo BIRD and continuously lose water ligands. After 30 s, the clusters have lost almost all water molecules, and ${\left({\text{H}}_{2}\text{O}\right)}_{m}{\text{Cl}}^{-}$, *m*= 3−6, is present in the mass spectrum.

**Fig. 2:**
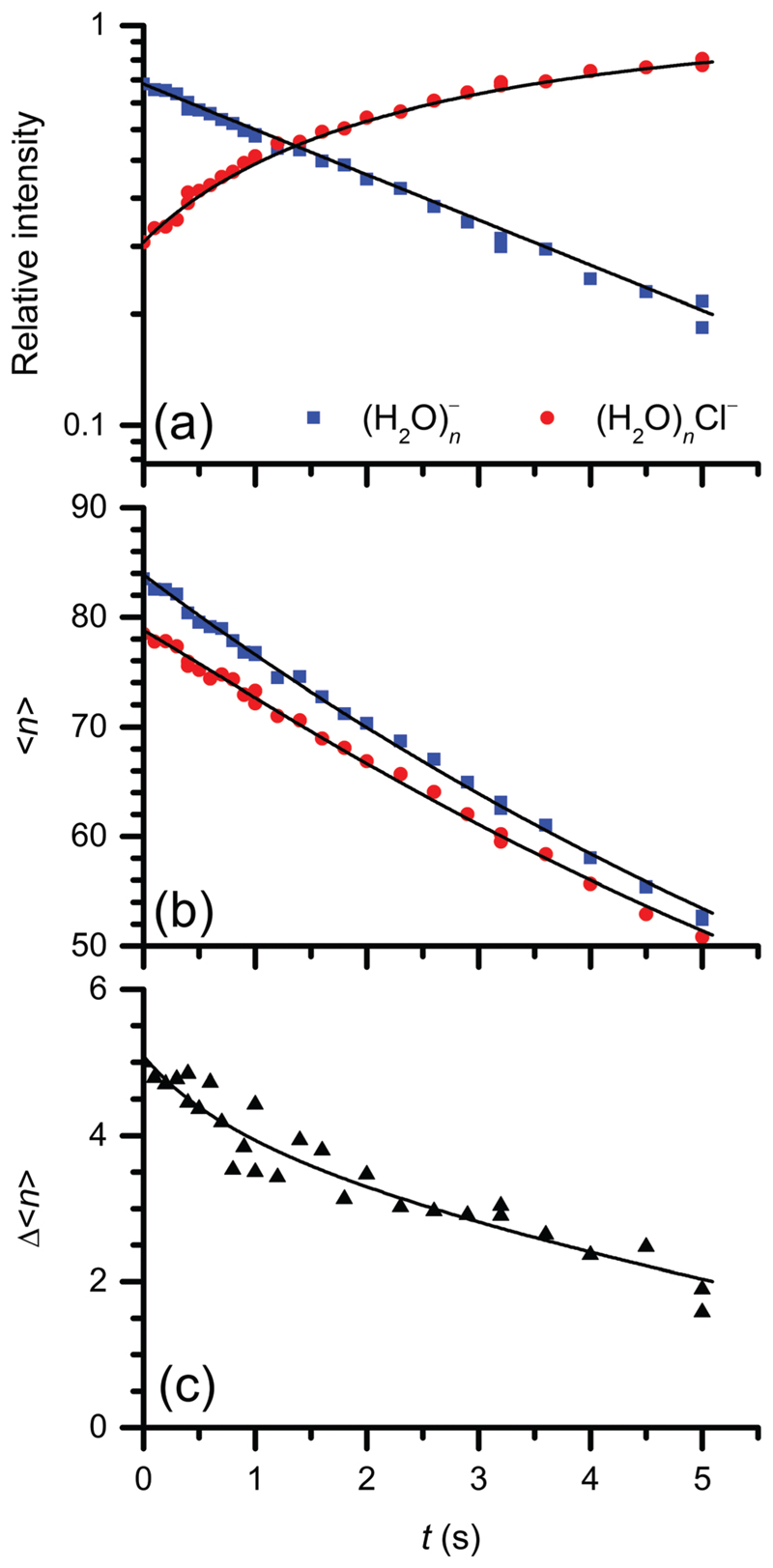
(a) Kinetic and (b and c) nanocalorimetric analysis of the reaction of CF_2_Cl_2_ with hydrated electrons (H_2_O)_*n*_^−^ at room temperature. Panel (a) represents the pseudo-first-order kinetic fit of (H_2_O)_*n*_^−^ (blue squares) as the reactant and (H_2_O)_*m*_Cl^−^ (red circles) as the product species. Panel (b) shows the fit of the cluster mean sizes for both species, and panel (c) illustrates the fit of their size difference (black diamonds).

To elucidate the reaction rate, the total intensities are plotted as a function of time. The intensities of reactant and product clusters in the mass spectra are summed over all cluster sizes and normalized. The reaction kinetics is quantitatively analysed for the first 4−8 s, depending on the initial cluster size. For *n*< 30, blackbody radiation induced electron detachment occurs, which interferes with the quantitative analysis.^33,44^ Thus all quantitative fits are stopped when the lower end of the cluster size distribution reaches *n*= 30. Fig. 2a shows the kinetic fit using a pseudo-first-order rate law. The resulting first order rate constant *k*_rel_[s^−1^] is converted to a pressure corrected absolute rate constant. A relative error of T25% is determined by the uncertainty of the pressure gauge. Since the CF_2_Cl_2_ pressure is a critical parameter for the absolute rate constant determination, the measurements were performed at different pressures repeatedly on different days to minimize any uncertainty. The results of each measurement are shown in Table 1. The measured experimental rate constants *k*_abs_ are compared with the calculated collision rates to determine the reaction efficiency. We have shown previously that average dipole orientation (ADO) theory,^45–48^ which describes the ion as a point charge, underestimates the collision rate of clusters with more than 10 water molecules (*k*_ADO_ = 6.1 × 10^−10^ cm^3^ s^−1^ for reaction with CF_2_Cl_2_). We therefore use models that account for the geometric size of the water cluster, in particular the hard sphere average dipole orientation, *k*_HSA_ = 1.0 × 10^−9^ cm^3^ s^−1^, and the surface charge capture model, *k*_SCC_ = 2.0 × 10^−9^ cm^3^ s^−1^.^49^ Earlier studies in our group indicate that the actual collision rate of ionic water clusters lies between the two models.^50–54^ The reaction efficiency can thus be determined using eqn (5).

(5) $$\Phi =2k_{\text{abs}}/\left(k_{\text{HSA}}+k_{\text{SCC}}\right)$$

Averaging over all experiments, we arrive at an absolute rate constant of $k_{\text{abs}}=8.6\pm 2.2\times 10^{-10}{\text{cm}}^{3}{\text{s}}^{-1}$, which corresponds to an efficiency Φ = 57 ± 15%. This means that about one out of two collisions is reactive.

The mass spectra reveal that the ${\left({\text{H}}_{2}\text{O}\right)}_{m}{\text{Cl}}^{-}$ ions have a slightly lower mean cluster size than the hydrated electrons ${\left({\text{H}}_{2}\text{O}\right)}_{n}^{-}$. This difference indicates that water molecules are lost due to exothermic reaction. Nanocalorimetry is employed, in which the average number of evaporated water molecules is determined.^30^ The mean cluster sizes for reactants and products as well as their difference were plotted as a function of time (Fig. 2b and c). A nanocalorimetric fit reveals that the reaction leads to the evaporation of 4.9 ± 0.9 water molecules. The energy required to evaporate a single water molecule from the cluster is Δ*E*_vap_ = 43.3 ± 3.1 kJ mol^−1^.^36,55^ The total energy release is almost identical to the absolute value of the room temperature reaction enthalpy, with minor corrections and a small contribution to the uncertainty.^30,50^ Then Δ*E*_raw_ can be calculated using eqn (6), which is converted to room temperature enthalpy Δ*H*_r_(CF_2_Cl_2_, 298 K) as detailed in the accompanying ESI.^†^

(6) $$\Delta E_{\text{raw}}=-\Delta N_{\text{vap}}\Delta E_{\text{vap}}=-214\pm 41{\text{kJ mol}}^{-1}$$

(7) $$\Delta H_{r}\left({\text{CF}}_{2}{\text{Cl}}_{2},298\text{K}\right)=-208\pm 41{\text{kJ mol}}^{-1}$$

The observed electron transfer reaction of CF_2_Cl_2_ with ${\left({\text{H}}_{2}\text{O}\right)}_{n}^{-}$ is strongly exothermic. To the best of our knowledge, no thermo-chemical data on the reaction of hydrated electrons with CF_2_Cl_2_ have been reported so far.

To compare the measured results with literature thermochemistry, we use the observed Δ*H*_r_ in combination with reaction enthalpies from the condensed phase to calculate the BDE of Cl– CF_2_Cl bond cleavage. The same approach was successfully used previously on SF_6_ as a benchmark for nanocalorimetry.^50^ BDE is calculated from established data, namely the hydration energy of the electron,^56^ the dissociation enthalpy of HCl,^57^ the solution enthalpy of gaseous HCl,^58^ and the ionization energy of the hydrogen atom.^59^ A thermochemical cycle including all reaction steps is summarized in Table 2. BDE as the enthalpy change of the overall reaction is calculated as the sum of the reaction enthalpies of the partial equations. This results in the C–Cl BDE of CF_2_Cl_2_, Δ*H*_C–Cl_(CF_2_Cl_2_, 298 K) = 355 ± 41 kJ mol^−1^.

In addition, the thermochemistry of the C–Cl bond cleavage of CF_2_Cl_2_ is derived by G4 calculations, in which BDE is obtained from the total enthalpies at 298.15 K. The calculated BDE amounts to 337 kJ mol^−1^. This lies within 18 kJ mol^−1^ of the experimental value. Both values agree within error limits with the published BDE of 346.0 ± 13.4 kJ mol^−1^ that was calculated from the standard enthalpies of formation.^60,61^

## Discussion

At first sight, the high exothermicity of reaction (4) may b surprising, given that the C–Cl bond dissociation energy of 355 T 41 kJ mol^−1^ is close to the electron affinity of the Cl atom, 348 kJ mol^−1^. However, the additional energy is supplied by the much stronger interaction of the Cl^−^ ion with the solvent environment compared to the hydrated electron. Essentially, hydration promotes DET.

**Fig. 3:**
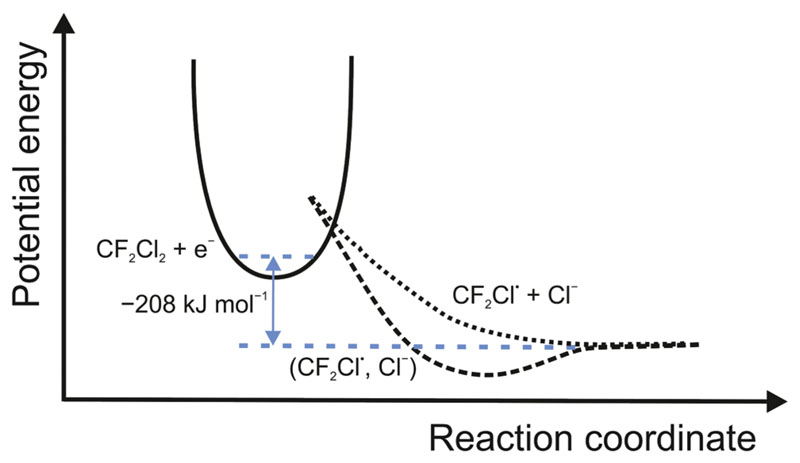
Schematic potential energy curves for sticky (dashed) or non-sticky (dotted) DET of a hydrated electron to CF_2_Cl_2_.

The mechanism of the reaction is straightforward. In the first step, the solvated electron moves to the σ* orbital of one of the C–Cl bonds, reducing the bond order from 1 to 0.5. Water molecules rearrange to solvate the incipient chloride ion, which further weakens the bond until it is broken and the CF_2_Cl^●^ radical is released. Whether a local bound minimum between the Cl^−^ and CF_2_Cl^●^ exists, *i.e.* whether the DET corresponds to a sticky DET in aqueous solution,^19^ cannot be determined on the basis of our experiments. We therefore depicted potential curves for both scenarios in Fig. 3. This mechanism can be discussed in connection with two other experiments mentioned in the introduction: the photodissociation of CF_2_Cl_2_^4,62^ and the DET mechanism to CFCs on ices.^5–9^

First, we discuss the photodissociation of CF_2_Cl_2_; it is a similar process to DET in the sense that the electron is promoted by a UV photon to the antibonding σ* orbital on one of the C–Cl bonds.^62^ In our recent study of this process on large water clusters^4^ we have not seen any evidence for free Cl fragments. Accompanying theoretical calculations revealed a halogen bond^63^ between Cl and O atoms of water molecules. Thus the CF_2_Cl_2_ molecule was bound to the ice nanoparticles with the Cl atoms oriented towards the cluster and the Cl fragment was caged by the cluster after photodissociation.^4^ This is consistent with the present observation that the Cl^−^ fragment of DET on water clusters remains with water clusters generating the observed ${\left({\text{H}}_{2}\text{O}\right)}_{m}{\text{Cl}}^{-}$ products, thus further supporting our previous proposal of halogen bonding between the CF_2_Cl_2_ molecule and water clusters. It also matches the observed efficiency of 57%, if one assumes that CF_2_Cl_2_ has to collide with the water cluster with the chlorine atoms facing the water network.

Concerning the DET mechanism to CFCs (and other molecules) on ices, it was proposed to proceed *via* so-called presolvated electrons with binding energies of about 1.3 eV below the vacuum level.^8,64–67^ The vertical detachment energy of solvated electrons in water clusters strongly depends on the cluster size. In the size range studied here, the VDE of ${\left({\text{H}}_{2}\text{O}\right)}_{n}^{-}$, *n*= 30–86, ranges from 1.46 eV to 2.00 eV.^68^ Extrapolation of cluster values to the bulk yields VDEs ranging from 3.3 eV to 4.0 eV in the literature.^68–71^ The VDE of bulk water is directly accessible from liquid jet measurements, where values of 3.3 eV have been reported.^72,73^ No adiabatic values are available for neat water clusters, but Donald *et al.* studied the hydration of free electrons in $\text{La}{\left({\text{H}}_{\text{2}}\text{O}\right)}_{n}^{3+}$, *n*= 42−160.^74^ From this study, they extrapolated a bulk hydration enthalpy of −1.3 eV for the electron, identical to the value suggested for the presolvated electron. Direct measurements of the adiabatic hydration enthalpy of an electron in bulk water usually refer to the absolute hydration enthalpy of a proton, which is not precisely known. Taking for example the value reported by Shiraishi *et al.* ^56^ referenced to Δ*H*_hyd_(H^+^) = −1090 kJ mol^−1^, which is the textbook standard, results in Δ*H*_hyd_(e^−^) = −172 kJ mol^−1^ or −1.8 eV. It should be noted that the thermochemical analysis presented here does not rely on the absolute hydration enthalpy of a proton or an electron, but only on their combined value, which should be very reliable. Since hydrated electrons in the excited state relax within 400 fs (*n*= 25) to 1 ps (bulk) to the electronic ground state,^75^ we can safely conclude that in our clusters, the electrons are in the electronic ground state. This implies that dissociative electron transfer to CF_2_Cl_2_ in the condensed phase does not require a presolvated state, which is in agreement with the earlier results from pulse radiolysis in bulk aqueous solution.^27^

## Conclusions

As previously observed in pulse radiolysis studies in bulk aqueous solution, our results with gas-phase hydrated electrons confirm that fully thermalized hydrated electrons in their electronic ground state are able to induce bond cleavage in CF_2_Cl_2_, analogous to the DET mechanism proposed for PSCs. The reaction is efficient and very exothermic. Nanocalorimetry combined with condensed phase literature thermochemistry yields thermochemical data that are consistent with literature values, as well as our own quantum chemical calculations. All these arguments together are consistent with the interpretation that CF_2_Cl_2_ undergoes DET in condensed aqueous environments, if thermalized hydrated electrons are present. Whether or not this mechanism is actually relevant for stratospheric ozone destruction is a different question, which cannot be answered on the basis of the present laboratory studies.

## † Supplementary Material

Electronic supplementary information (ESI) available: Conversion of ΔE_raw_ to ΔH_298K_. See DOI: 10.1039/c6cp01976e

Supporting Information

## Figures and Tables

**Table 1:** Kinetic and nanocalorimetric analysis of each data set

(H_2_0)_*n*_^−^	*n = p*(CF_2_Cl_2_)/10^−9^ mbar	k_abs_/10^−10^ cm^3^ s^−1^	k_HSA_/10^−9^ cm^3^ s^−1^	k_scc_/10^−9^ cm^3^ s^−1^	k_ADO_/10^−10^ cm^3^ s^−1^	*Φ* (%)	ΔN_vap_	ΔE_raw_/kJ mol^−1^
31–47	7.2	7.1	0.9	1.8	6.2	53	4.99	−216
30–47	11	6.5	0.9	1.8	6.2	48	5.39	−233
36–50	4.7	6.6	0.9	1.9	6.2	47	6.02	−261
34–54	9.0	7.5	0.9	1.9	6.2	54	4.18	−181
35–58	8.1	7.4	0.9	1.9	6.2	53	3.41	−148
50–82	9.0	9.4	1.0	2.1	6.0	61	5.21	−226
51–82	11	10	1.0	2.1	6.0	65	5.13	−222
51–84	11	12	1.1	2.1	6.0	75	6.92	−300
51–84	8.3	9.5	1.1	2.1	6.0	59	2.57	−111
46–86	9.3	10	1.0	2.1	6.0	65	5.57	−241
Average	–	8.6	1.0	2.0	6.1	57	4.94	−214

**Table 2:** Thermochemical cycle for the Cl–CF_2_Cl BDE

Reaction	ΔH_r_ (298 K)/kJ mol^−1^	Ref.
H^+^(g) + e^−^(g) → H^+^(aq) + e^−^(aq)	−1261.9	56
H^+^(aq) + Cl^−^(aq) → HCl(g)	74.48	58
HCl(g) → H(g) + Cl(g)	431.58	57
H(g) → H^+^(g) + e^−^(g)	1318.4	59
CF_2_Cl_2_(g) + e^−^ (aq) → CF_2_Cl(g) + Cl^−^(aq)	−208	Our work
CF_2_Cl_2_(g) → CF_2_Cl(g) + Cl(g)	355	Sum
