# Supplementary material for: The reaction of CF2Cl2 with gas-phase hydrated electrons†
Source: Phys Chem Chem Phys. Author manuscript; Available in PMC 2020 Nov 8. (PMC7116337; doi:10.1039/c6cp01976e)
Supplement: Supporting Information [file EMS102277-supplement-Supporting_Information.pdf]

Electronic Supplementary Material (ESI) for Physical Chemistry Chemical Physics.
This journal is © the Owner Societies 2016

Supporting Information for article

Reaction of CF_2Cl_2 with Gas-Phase Hydrated Electrons

Jozef Lengyel,*† Christian van der Linde,† Michal Fárník‡ and Martin K. Beyer*†

† *Institut für Ionenphysik und Angewandte Physik, Leopold-Franzens-Universität Innsbruck, Technikerstraße 25, 6020 Innsbruck, Austria.* E-mail: jozef.lengyel@uibk.ac.at; martin.beyer@uibk.ac.at
‡ *J. Heyrovský Institute of Physical Chemistry v.v.i., Czech Academy of Sciences, Dolejškova 3, 18223 Prague, Czech Republic.*

Conversion of ΔE_{raw} to ΔH_{298K}

Temperatures in the experiment:

$(H_2O)_n^-$	+	CF_2Cl_2	→	$(H_2O)_mCl^-$	+	$CF_2Cl^\bullet$	+ $(n-m)$ H_2O
90 - 120 K		298 K		90-120 K		120-210 K	

The internal temperature of water clusters is a result of radiative heating and evaporative cooling. We assume here a value of 90 – 120 K, which corresponds to the region where the solid-to-liquid phase transition occurs.[1] The neutral reactant is at room temperature, equilibrated in collisions with the surfaces in the UHV region of the mass spectrometer. The neutral products, including the evaporating H_2O molecules, will have an internal energy distribution that corresponds to the internal temperature of the cluster after the reaction. The reaction enthalpy is heating the cluster with 214 kJ mol^{-1}. Setting n = 50, there are about 6n = 300 low-lying vibrational degrees of freedom which correspond to the translational and rotational degrees of freedom of the free water molecules. If we assume that these are thermally populated, we have 0.7 kJ mol^{-1} per degree of freedom. A fully populated vibrational degree of freedom contains RT internal energy, therefore the 0.7 kJ mol^{-1} per degree of freedom correspond to a temperature increase of 86 K. Of course, the cluster immediately responds with evaporative cooling, therefore we do not know the exact temperature at which each neutral molecule evaporates. In addition, instead of increasing the temperature, the cluster may convert the additional energy into latent heat by breaking hydrogen bonds. Since a detailed modeling of all these aspects goes beyond the scope of the present work, we give the conservative range above.

From nanocalorimetry, we obtain ΔE_{raw} at these conditions:

$$\Delta E_{raw} = -\Delta N_{vap}\,\Delta E_{vap} = -214 \pm 41 \text{ kJ mol}^{-1}$$

Corrections for ΔH_{298K}:

The difference in heat capacity of a hydrated electron compared to hydrated chloride is unknown. However, the three vibrational degrees of freedom of the Cl^- ion oscillating in the cluster is an upper limit. If these low-lying modes, we have a contribution to the heat capacity of 3R. Since the heat of the reaction has to provide the energy to populate these modes, this effect reduces the exothermicity, therefore the correction has a positive sign. Since the correction lies somewhere between 0 and 3 RT, we suggest:

$\Delta(\Delta H)_C$ = (1.5 ± 1.5) [R(298 K – 105 K)] = 2.4 ± 2.4 kJ mol^{-1}

The same argument applies to the neutral reactant and product. For ΔH_{298K}, the $CF_2Cl^{\bullet}$ must be heated to 298 K. The heat capacity of a rigid rotor without thermally populated vibrational levels is 3R. The correction therefore amounts to:

$\Delta(\Delta H)_{CF2Cl}$ = 3R (298 K – (165 ± 45 K)] = 3.3 ± 1.1 kJ mol^{-1}

In summary, we obtain with Gaussian error propagation:

$\Delta H_{298K} = \Delta E_{raw} + \Delta(\Delta H)_C + \Delta(\Delta H)_{CF2Cl}$ = −214+2.4+3.3 ± sqrt(41^2+2.4^2+1.1^2) kJ mol^{-1} = −208 ± 41 kJ mol^{-1}

References

(1) Hock, C.; Schmidt, M.; Kuhnen, R.; Bartels, C.; Ma, L.; Haberland, H.; von Issendorff, B. Calorimetric Observation of the Melting of Free Water Nanoparticles at Cryogenic Temperatures. *Phys. Rev. Lett.* **2009**, *103* (7), 73401. DOI: 10.1103/PhysRevLett.103.073401.
